# Solving the riddle by puzzling it out: overcoming the challenges of harm–benefit analysis by assembling a compound model and practice-oriented tool

**DOI:** 10.3389/fvets.2025.1593954

**Published:** 2025-12-11

**Authors:** Dominik Hajosi, Herwig Grimm

**Affiliations:** 1 Department of Interdisciplinary Life Sciences, Messerli Research Institute, University of Veterinary Medicine Vienna, Vienna, Austria; 2 Institute of Comparative Medicine, Columbia University, New York, NY, United States

**Keywords:** harm-benefit analysis, guidance document, decision-making, ethical review, animal research ethics committees

## Abstract

The practical application of harm–benefit analysis (HBA), a requirement mandated by European Union Directive 2010/63/EU, has been extensively examined. However, reviewing bodies can access only sparse and often incomplete HBA guidance. This study develops a composite HBA method composed of the relevant and useful elements from current guidance documents and provides instructions on conducting such an analysis. It redefines HBA approaches, eliminating redundant aspects while meeting the Directive’s requirements. This novel compound HBA method includes a clear procedural methodology to guide project applicants, reviewing bodies, and authorities in transparent and coherent decision-making.

## 1 Introduction

Harm–benefit analysis (HBA) was introduced as a central element of project evaluation in European Union (EU) Directive 2010/63/EU (hereafter “the Directive”), which is the primary framework regulating the protection of animals used for scientific purposes in European Union (EU) Member States (1). An HBA supports decision-making on whether animal use is justified and can be approved. As such, a project applicant must demonstrate not only that the “3Rs” (replace, reduce, and refine) have been taken into consideration to minimize animal suffering and that foreseeable harm cannot be avoided (1) but also that the project’s value in terms of expected outcomes and benefits balance or outweigh the harm inflicted on animals.

Article 38 of the Directive states that as part of project evaluation, an HBA must be carried out “to assess whether the harm to the animals in terms of suffering, pain and distress is justified by the expected outcome taking into account ethical considerations, and may ultimately benefit human beings, animals or the environment” (1). However, while the use of HBA has been extensively examined, the concepts supporting its application remain fragmentary rather than comprehensive (2–6).

Several European countries have published guidance documents (GDs) at both the national and regional levels to support the application of HBA during the project review process (2). While the key elements of HBA (which we refer to as domains) are set out in the Directive’s text, its practical implementation remains unclear (2, 7). Although the Directive has been a legal requirement in EU Member States for over 10 years, with the mandate that project evaluation “shall be transparent” (1), a comprehensive HBA method has not been developed and differences in its status exist (2, 8). Guidance, if available, often provides only vague directions that create inconsistencies in HBA implementation (2). Therefore, the decision-making process during project evaluation can often experience significant challenges (9), leading to the use of incoherent and nontransparent criteria and the inconsistent treatment of project applicants by reviewing authorities. Furthermore, the extent of operationalizing HBA among Member States is often unclear (8, 10–13). Indeed, only 6 of the 30 European countries investigated have produced GDs to support HBAs during project evaluation (2).

This lack of explicit and comprehensive methodological guidance for and inconsistent application of HBAs is problematic for several reasons. First, if different criteria and standards are applied to project evaluation by different reviewing bodies, comparable projects are at risk of being evaluated differently and unequally (2, 3, 9, 14). Second, it can undermine the basis, reasons, or justifications used to approve or reject projects (14, 15), making reviewing authority decisions hard to understand or nebulous and contradicting the Directive’s “transparency” mandate (1). Third, it can create frustration among the project applicants, competent authorities, and reviewing bodies, complicating the evaluation process—applicants lack relevant criteria to address, while reviewers and authorities may become dissatisfied with the applicant’s rationale, arguing that their review criteria have not been effectively met or that a project’s application does not align with their (uncommunicated) expectations. Fourth, there is a risk that the evaluation criteria applied during reviewing committee work may exceed what is legally required by the Directive (16). These reasons reflect the fractured structure of HBA and the challenges experienced in its application, making it difficult to achieve given the fundamental standards imposed by the rule of law.

Efforts to overcome these challenges have led to the development of various HBA methods. Numerous HBA methods have been described, with each offering advantages but also having significant issues (6, 17–19). While these methods appear structurally different, they share a critical issue: none has been fully implemented. There could be different reasons for this issue—as described in the literature (3)—but it is primarily owing to uncertainty over which method is effective and should be applied. The two main HBA methods are the discourse model (DM) and the metric model (MM) (3). In summary, the DM approach uses a discursive format for the HBA, in which the reviewing committee members advising the competent authority discuss the harms and benefits of the proposed project and make a decision. This approach is often followed by Member States and yet remains relatively unstructured (3, 9, 20, 21). The lack of standardization also risks inconsistent decision-making owing to committee member biases in terms of preferences, views, knowledge, and/or expertise.

In contrast, the MM approach provides a more explicit and transparent methodology. It involves at least four steps (3): (1) the harms and benefits are operationalized using measurable criteria, (2) specific weightings are attributed to the harms and benefits, (3) a methodology to assess the harms and benefits is developed, and (4) a procedure is established to evaluate the harms and benefits against each other. Thus, the MM approach enables a decision on a project to be made based on a fixed and standardized metric or even an algorithm. While this model offers a structured and standardized method, it is not often used because it is considered too technical and lacks the flexibility needed for different and innovative research areas. Furthermore, who is ultimately accountable for and can legally enact this methodology remains unclear (3).

In addition, the advantage of the MM approach also has a cost. In contrast to the DM approach, which is considered too flexible for coherent project evaluation, the MM approach is regarded as too rigorous and mechanical. Grimm et al. (3) compared both models and summarized their challenges. Consequently, the suggested merger of the two approaches may provide a solution, combining their positive elements and discarding their negative features (3). This could use the strengths of both models, leading to a practical and conducive decision-making tool. However, this composite approach has yet to be developed; to develop and provide such a tool is the aim of this study.

It is important to note, that before conducting an HBA of any kind in this context, a project must first demonstrate that it adheres to the 3Rs (22). Only once the 3Rs have been met can an HBA be meaningfully initiated. This sequential procedure is crucial as applying the 3Rs identifies legally justifiable (although not yet justified) harms within a project (23). As such, a combined HBA approach requires the application of the 3Rs as a prerequisite. Similarly, the 3Rs may provide a resource for reviewing bodies to address preliminary questions regarding a project’s application in a more structured manner, supporting more robust decision-making, which is then reinforced by discussions through the combined HBA approach or by committee member voting.

As such, as proposed by Grimm et al. (3), such a combined tool aims to navigate between the MM and DM approaches by: (1) making HBA less technical and more practical, bringing it closer to the DM approach; and (2) incorporating standardized criteria that committee members use during deliberations, aligning HBA with the well-structured and guided process used in the MM approach. Hence, this study develops an HBA approach that combines these two existing methodological concepts into a merged HBA model—the compound model (CM).

**Figure 1:**
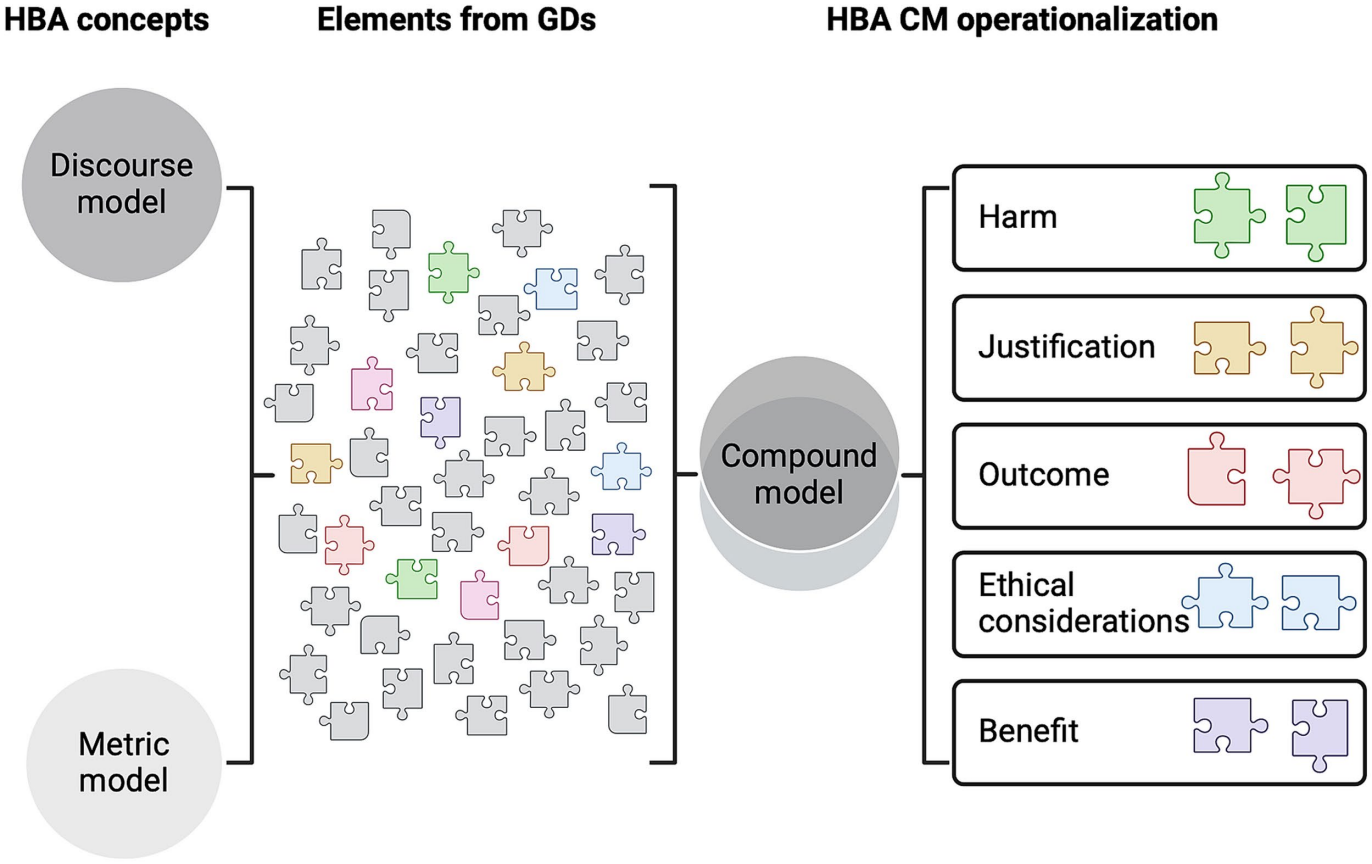
Overview of the steps to create a compound model for harm–benefit analysis. Compound model (CM); guidance documents (GDs); harm–benefit analysis (HBA).

This offers a potential solution to some of the main problems experienced during the project evaluation process. It fills the current methodological black hole with a transparent approach that aligns with the Directive’s requirements and meets the HBA domains discussed in recent literature (2) (i.e., harm, justification, outcome, ethical considerations, and benefit, as described within the Directive). Therefore, this study considers what project applicants should be asked regarding the HBA requirement and how committees should handle the information provided by the applicant. The study proposes an HBA method composed of various elements that provide a tool to effectively address HBA as a legal requirement (see Figure 1).

In summary, the primary objectives of this study are: (1) to analyze the individual HBA domains by focusing on their roles within the MM and DM approaches as part of developing the CM, (2) to establish a transparent methodology for undertaking a compound HBA based on current GDs, and (3) to provide clear, practical guidance on this novel method. We hypothesize that the CM provides a practical tool that meets the requirements of the Directive and offers transparent guidance for the project evaluation process. The research question guiding these objectives is as follows: learning from existing GDs, how can the Directive’s HBA domains be effectively integrated into a compound HBA model that meets the Directive’s requirements?

## 2 Materials and methods

The materials used in this study comprised the HBA domains set out in the Directive’s HBA requirement and current literature (2) (see Section 2.1), available HBA GDs [as discussed in (2)] (see Section 2.2), and existing HBA methods (i.e., versions of the DM and MM approaches and their respective issues) (see Section 2.3).

### 2.1 The directive’s harm–benefit analysis domains

Article 38 of the Directive sets out the mandatory parts of a project evaluation, including the HBA (1). Existing literature has shown that the HBA requirement can be divided into the following core domains: harm, outcome, benefit, justification, and ethical considerations (see Table 1) (2). This study is organized around these domains because they must be integrated into any HBA method for it to comply with the Directive. Therefore, these domains provide the foundation and framework for this study.

**Table 1:** The Directive’s harm–benefit analysis requirement and related domains.

The Directive’s HBA requirement
“A harm-benefit analysis of the project, to assess whether the harm to the animals in terms of suffering, pain and distress is justified by the expected outcome taking into account ethical considerations, and may ultimately benefit human beings, animals or the environment” (1).

HBA domains	Description within the HBA requirement
Harm	“harm to the animals in terms of suffering, pain and distress”
Justification	“is justified”
Outcome	“by the expected outcome”
Ethical considerations	“taking into account ethical considerations”
Benefit	“may ultimately benefit human beings, animals or the environment.”

### 2.2 Harm–benefit analysis guidance documents

This study considered GDs identified in existing literature (2). Seven GDs were included that methodologically operationalize HBA (see Table 2). The CM approach extracted the parts of these GDs considered relevant and useful to the practical application of HBA and combined these established elements into a cohesive framework. Wherever an apparent omission or concern regarding an element’s operationalization was identified, a solution was proposed from outside these existing GDs. This ensured that the CM remained comprehensive and could be implemented without unexplained uncertainties.

**Table 2:** Overview of guidance documents.

Guidance documents	
Country with a national *(N)* or regional *(R)* guidance document	Abbreviation
EU/EC (overarching framework)	HBA-EU
Austria *(N)*	HBA-AT
Belgium *(R)*	HBA-BE
France *(N)*	HBA-FR
Netherlands *(N)*	HBA-NL
United Kingdom *(N)*	HBA-UK
Switzerland *(N)*	HBA-CH

### 2.3 Relevant harm–benefit analysis methods

The DM and MM approaches were the primary HBA models considered. While we have also applied methodological insights from other studies, we do not discuss individual HBA methodologies for the following reasons: (1) this has already been undertaken elsewhere in considerable detail (6, 17); (2) our interest lies in the methodological differences and the respective positive and negative aspects that can be identified and used (or avoided) in the CM approach; and (3) to make the CM approach feasible for a variety of applications, we have structured it following the Directive’s requirements rather than an HBA method proposed in the literature. As such, brief definitions of each model, including their advantages and disadvantages, are provided—these models are discussed in detail elsewhere (3).

In the DM approach, the harms and benefits, including the evaluation process used for these two domains, are assessed through an open dialogue among the committee members. The committee is responsible for evaluating the harm to which animals would be subjected as well as the project’s benefits without using predefined criteria. The decision on an individual project proposal is reached through a process of reasoning, active participation, and engagement of all of the committee members. Typically, the recommendation for approval or rejection is based on a majority vote.

However, the rules governing this process are not well defined—if at all. The DM approach benefits from its relatively loose structure, providing flexibility and contextualizing the arguments for and against. Nevertheless, a disadvantage of this process is that such project evaluation is prone to inconsistencies within and between committees, which is largely due to the lack of standardization. A fair and transparent discussion that leads to a reasonable and objectively comparable outcome is the ideal scenario but also a demonstrable concern (3, 9).

In comparison, the MM approach directly relates the harms and benefits by defining measurable dimensions. These dimensions can then be placed on a symbolic scale and evaluated against each other following a set procedure. One of the aims of the MM method is to take a more categorical, algorithmic approach. Ideally, the MM method would be applied as a tool as part of a systematic HBA. However, following a strict arithmetic approach can hinder broader deliberations and open reflections, given its lack of flexibility (3). A summary of the disadvantages of current HBA methods, as described by Grimm et al. (3), is provided in Table 3.

**Table 3:** The harm–benefit analysis problem list (3).

Problem list	
Metric model	Discourse model
Unclear accountability: who is accountable for decisions based on predefined criteria?	Accountability: the reviewing committee is the accountable entity as it deliberates on the project.
Predefining criteria: primarily for the harm and benefit domains.	Criteria are nontransparent.
Weighting of criteria: what is the relative weight of the predefined criteria?	Committee composition shapes the outcome: the differing professional and social backgrounds of the committee members influences deliberations and ultimately the decision made.
Operational assessment: (to what extent) are the criteria fulfilled?	Deliberations get out of focus: a lack of guidance broadens discussion to non-HBA-related assessments.
Developing a procedure: the procedural goal is to reach a decision based on assessing harms and benefits.	Limitations in standardization: how are the procedural steps and outcomes standardized efficiently?
Likelihood of reaching an agreement on the methodology: a relatively low chance of reaching a consensus on a uniform method among differing stakeholders and/or experts.	Lack of methodology raises questions: While input from all committee members must be considered, how is it effectively considered during deliberations? How are deliberations kept within reason and do not become unsuccessful? The operational challenge of being consistent: how are deliberations standardized?
Diversity of projects: a standardized approach does not reflect the complexity and diversity of projects, risking bias ex ante and inappropriate outcomes by applying standardized criteria or template.	Diversity of projects: contextual factors may influence deliberations and outcomes for certain projects.

## 3 Results

The following section describes the HBA domains in a sequence, highlighting the order in which they should be approached in the CM. These domains were analyzed using the operative tools identified in the GDs and existing HBA methodologies, enabling the CM method to be developed reflectively. Relevant positive and negative aspects were identified and resolved while maintaining the focus on combining the DM and MM approaches into the CM method. As such, the findings condensed promising methods to effectively address the HBA domains, thereby enabling the CM to be assembled.

### 3.1 Outcome domain: knowledge gain as a prerequisite

The outcome domain is evaluated first because this domain represents a fundamental prerequisite in the CM. An HBA is futile if a meaningful outcome is not ensured. A recurring issue with HBA is a lack of clear definitions for the benefit and/or outcome domain. While the harm domain is extensively defined within the Directive and available GDs (2), this is not the case for the remaining domains—particularly the domain dealing with expected results of a project.

A major inconsistency has been the differentiation between outcome and benefit. Recent literature (2, 24) has shown that these terms mean different things, and differentiating between them is important if these domains are to be effectively translated in HBA, particularly in a CM approach. As such, we differentiate between these two elements and consider the outcome domain and the benefit domain as separate but linked domains. The outcome domain can be defined as the answer to the question: what will I know after carrying out the project? In contrast, the benefit domain represents the answer to the question: what do I (practically) want to achieve in the world based on the project’s outcome?

For the CM, the outcome domain is the prerequisite for the analysis of harms and benefits because the outcome is the basis for the expected benefits. Therefore, the first step in the CM is to ensure that a relevant outcome is at stake. The subsequent realization of the other HBA domains is only plausible if the outcome domain has been effectively addressed (i.e., the scientific purpose). Without an achievable scientific purpose, one of the core criteria controlling animal use is not met. Any project (consisting of procedures) must have a scientific purpose; the Directive clearly defines a procedure as “any use, invasive or non-invasive, of an animal for experimental or other scientific purposes, with known or unknown outcome, or educational purposes” (1).

The outcome—the result of achieving a particular scientific purpose—of a project is a crucial condition for achieving benefits; therefore, the outcome must be both relevant and attainable to justify the harm inflicted on animals and pursue the anticipated benefits. However, knowledge gain is not identical to the generation of a benefit. A benefit can be gained owing to acquired knowledge or through a practical, real-world impact. The terminological inconsistency between outcome and benefit is often overlooked, and the available GDs have typically not highlighted the difference between them (2). Hence, the operational steps needed to incorporate an analysis of outcomes are often vague.

To our knowledge, this study represents the first attempt to integrate the outcome domain as a distinct component within the Directive’s HBA framework. Our CM method includes a systematic approach to incorporate this domain. This involves the following steps: (1) describing the knowledge gain, (2) assigning the project to an outcome stream (i.e., one of the Directive’s project purposes as set out in Article 5 (1)), (3) characterizing the outcome domain, and (4) subjecting the project to a rigorous evaluation to examine its scientific rigor and the key methodological components necessary to achieve the stated outcome.

This approach seeks to avoid overestimating a project’s expected benefit while confirming that its outcome is relevant to a particular research area. To adequately address this domain, the applicant must provide detailed and comprehensive information to enable scientific evaluation of its intended outcome and the likelihood of achieving it. Evaluation of the benefit dimension follows later in the process (see Section 3.3).

Scientific evaluation of the outcome domain can be achieved in two ways: (1) funding agencies can conduct a merit review, assessing a project’s scientific merit and the significance of its outcome through a peer review; and (2) the reviewing committee can assess a project’s scientific merit using subject matter experts who consider whether a project is scientifically robust and sound and whether its outcome is relevant to its research area.

**Figure 2:**
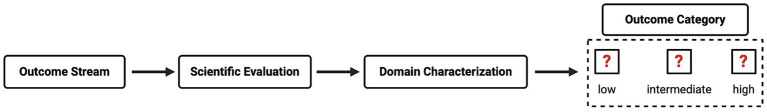
Outcome domain evaluation.

In summary, a project’s outcome domain is the first step in the CM process, which can be addressed by evaluating the domain’s characterization and quality. The reviewing committee can then categorize the outcome domain as either low, intermediate, or high, reflecting the impact, relevance, and likelihood of the outcome domain (see Figure 2).

### 3.2 Harm domain: building on longstanding practice and regulatory clarity

The harm domain represents the next step in the CM method and is another key dimension of the HBA process. Evaluating the harm inflicted on animals is a longstanding practice (6). Among the HBA domains, the harm domain has undergone the most rigorous and comprehensive examination and development. A project’s harm can negatively impact not only animal welfare but also the validity of its results (25); therefore, advisory groups have developed approaches to assess the harm caused to animals (2, 6, 26). The Directive introduces the concept of a severity classification, and its Annex VIII provides examples classifying the severity of individual procedures. A project’s severity category is determined by the individual that is most severely harmed (1), reflecting the Directive’s individualistic nature; as such, it is not the number of animals that is most important, but the individual that is most harmed. Although still debated, prioritizing the individual harm over the number of animals being harmed, leads to the common understanding of the 3Rs, which favor more animals being less harmed over fewer animals being more severely harmed. This aspect is crucial in the CM process, with the number of animals only included as a secondary modulating factor (MF).

There was a consensus among all of the GDs examined by Hajosi and Grimm (2) that the harm domain can be adequately operationalized only if it is primarily assessed by its severity and additional MFs. While Annex VIII provides a reference to assess and categorize harm inflicted on animals during a project, the available GDs show that a simple severity categorization of a procedure is insufficient and that certain MFs must also be considered to ensure a complete and comprehensive harm domain assessment.

As described in the Belgian and British GDs (27, 28), harm can be further differentiated into project-related harm (i.e., any harmful intervention directly affecting an animal due to a procedure, such as a surgical intervention) and contingent harm (i.e., harm attributable to the specific scientific use of an animal, such as housing it in a contained enclosure). These harms can have immediate and delayed effects. In addition, MFs can influence an animal’s experience—positively or negatively—during a project and, therefore, the overall harm domain.

Similarly, MFs can be differentiated into primary and secondary elements, providing a more precise and nuanced evaluation of harm. Primary MFs have an immediate impact on an individual animal’s experience during a project, such as pain relief measures or a procedure’s duration. In contrast, secondary MFs indirectly influence a project’s harm domain and are related to project parameters, such as the selection of a less sentient animal species or the total number of animals used. As such, secondary MFs do not directly cause harm in a predictable manner and are not considered determinants of any baseline severity. A clear distinction between primary and secondary MFs enables a more tailored assessment of the harm domain and its justification process.

The Belgian and European Commission’s GDs provided a comprehensive list of MFs (27, 29). These are defined and categorized as primary or secondary MFs in Table 4.

**Table 4:** Overview of modulating factors.

MFs in Belgian and European Commission’s GDs	Primary MFs	Secondary MFs	Explanation for assignment to primary or secondary MF
Methods to control adverse effects	✓		These relate to the direct mitigation of a procedure’s impact on an individual animal.
Procedure frequency and duration	✓		Length and intensity directly affect an animal’s experience of harmful procedures.
Severity level	✓		This directly reflects an animal’s experience and the immediate evaluation of potential pain, suffering, and/or distress.
A procedure’s termination and endpoints	✓		Methods and time points for a procedure’s termination are determined (i.e., an end (direct intervention) to an animal experiencing harm).
Overall life experience	✓		A cumulative effect that shapes an animal’s overall experience (e.g., the frequency of usage, an animal’s fate (death, reuse, rehoming, etc.), habituation procedures, etc.).
Health status	✓		This can impact an animal’s ability to cope with procedures or can directly cause harm.
Housing details	✓		Enclosure provisions, enrichment options, and social- versus single-housing modality can directly impact an animal’s well-being and the harm domain.
Care and monitoring regime	✓		The quality, frequency, and routine (husbandry) of care provision and health monitoring measures can have immediate effects on an animal.
Competence of animal care and research personnel	✓		Expertise and acquired or trained skills influence the care provided to animals and the quality of the procedures conducted.
Animal origin		✓	An animal’s origin can indirectly affect it owing to acclimation or habituation issues and/or pre-existing health conditions.
Transportation		✓	Transport-induced health issues or stress (related to transportation frequency and distance) can indirectly affect an animal before any procedure.
Genetic modifications and their impact		✓	An animal’s physiology can be indirectly influenced by phenotypic changes caused by genetic modifications.
Species		✓	Selection of certain animal species can indirectly influence the harm domain (e.g., using species known to be more or less sentient or with differing cognitive abilities).
Total number of animals		✓	An indirect factor that may influence the overall harm assessment.
Duration in proportion to an animal’s lifespan		✓	Duration can indirectly influence the harm domain as its effect emerges cumulatively over the course of a project in relation to an animal’s lifespan.

However, these MFs are not considered equally relevant to the CM approach. Consequently, the CM applies a more nuanced approach that incorporates the “total number of animals” MF. As individual animals used in a project may experience different procedures, it is important to account for the proportion of animals within each severity category. This provides a more detailed view of a project by providing information on how animals are categorized into specific severity categories [see Austrian GD (30)].

Table 5 provides an example of this approach for illustration. A cancer study investigating tumor induction and development includes a protocol involving a total of 325 rodents. Within this group, subsets of animals are expected to experience tumor induction with varying levels of disease progression. Of the 325 animals, 25 animals (Group A) will undergo a non-recovery procedure, while 50 animals (Group B) will receive tumors and experience lethal disease progression, placing them in the highest severity category. A further 100 animals (Group C) will experience moderate pain and distress during tumor induction, while 150 animals (Group D) are not expected to experience any adverse clinical effects. A detailed breakdown of these groups clarifies the proportion of animals within each of the four severity categories (non-recovery, mild, moderate, and severe). By presenting these values as percentages for each group, it is possible to infer the general severity trend across all of the groups.

**Table 5:** Allocation of the study groups to severity categories for the illustrative cancer study.

Study group	Number of animals	Percentage of animals	Severity category
A	25	8	Non-recovery
B	50	15	Severe
C	100	31	Moderate
D	150	46	Mild

While severity distribution within a project provides a clear picture of the total harm, the Directive requires that the severity category is based on the individual animal experiencing the most harm. Therefore, following the Directive’s requirements, the severity classification represents the harm domain’s main aspect, and the primary and secondary MFs are differentiated. While the primary MFs have a direct impact on the severity classification, the secondary MFs do not. This has substantial implications, including the number of animals having only a secondary role, which may appear problematic. However, this approach aligns with the Directive’s scope of protection in which only the individuals’ pain, suffering, and distress are defined as harm and must be justified. The animals’ lives are not protected, and the loss of life does not count as harm (rather, it is a refinement measure). As such, harm factors only in the HBA when animal use is associated with experiential harm, and the most harmed individual animal sets the level in the severity classification. This logic is also applied to our development of the CM method.

Therefore, the absolute number of animals is not considered a primary aspect because the Directive focuses on the severity experienced by an individual. In addition, from the project’s perspective, the number of animals is either correct or incorrect rather than high or low and is not something that can be readily changed—if too few animals are used, the scientific objective cannot be achieved, while if more animals are used than needed to meet the scientific objective, the project would not comply with the Directive’s 3Rs requirement. Moreover, the number of animals to be used should be determined by statistical evaluation and expert input, whenever possible, preferably by a power analysis. Therefore, the number of animals is an aspect that cannot be altered freely—decreased or increased—without having a significant negative impact (too little power or too many animals). Thus, the number of animals is either correct or incorrect and the total number of animals can only have a secondary role (i.e., secondary MF). An incorrect number of animals would be a failure criterion.

A third reason for not including the number of animals as a primary factor is that it is difficult to obtain agreement on what a high or low number of animals should be. Some research areas use more animals on average than others. When seeking to address the issue of what a high or low number of animals is, research areas that use fewer animals would have an advantage over those that use more—despite such research areas being prevented from using fewer animals because this would hinder their ability to generate the anticipated outcome. In summary, the total number of animals is highly problematic and represents too coarse an indicator, and its influence on an HBA should be limited. Therefore, it is included as a secondary MF only.

Several GDs included practical methods to enhance the harm domain assessment; for example, the Austrian GD requires that a project applicant provide detailed responses about the expected harm, including the number of animals in each severity category. Similarly, the Belgian GD includes targeted questions within the project application and requires that the applicant describe the relevant factors comprising the harm domain. Direct questions can be useful in identifying harms and MFs associated with a proposed project. Therefore, to perform a comprehensive harm domain evaluation using the CM approach, the following steps must be completed: (1) identify all harms that individual animals within their respective study groups are subjected, (2) categorize the project’s severity based on the most severely affected individual animal, and (3) characterize the harm domain by considering secondary MFs and animal numbers and/or percentages that influence the individual harm experienced.

Identifying all harms associated with a project involves classifying harms by severity for all study groups within the project; this ultimately represents the key factor that determines the project’s severity classification. Through this approach, both the immediate and delayed effects of a procedure are captured, providing a detailed account of the harm that would be inflicted during a project. This enables the identification of an appropriate severity classification that is based on the accumulated harms of the most harmed individuals within the study groups. Furthermore, the MFs that influence the harm experienced—either exacerbating or alleviating harm—must be assessed. Using Annex VIII of the Directive, together with detailed insights from the European Commission’s and Belgian GDs, ensures that the relevant MFs are evaluated.

**Figure 3:**
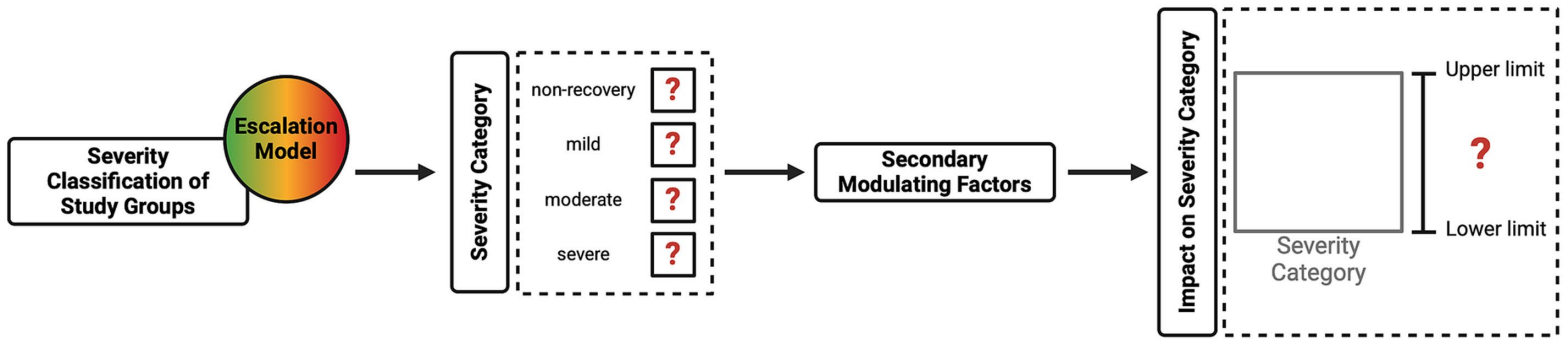
Harm domain evaluation.

**Figure 4:**
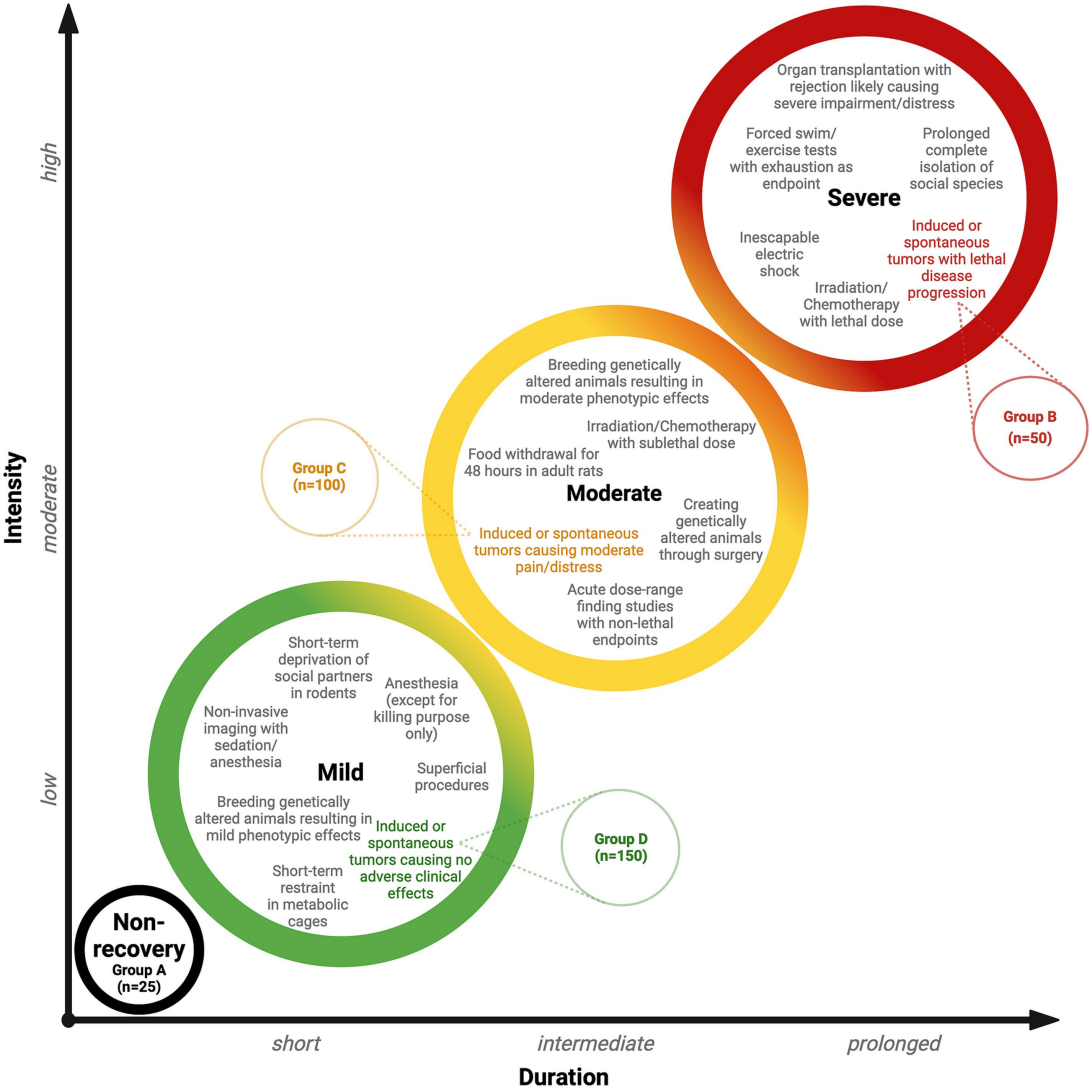
Escalation model. Examples of procedures are described within each colored circle (severity category) and are taken from Annex VIII of the Directive. The model uses the illustrative cancer study described previously and in Table 5 and shows the severity categories and the allocation of the study groups (see Table 5 for groups A–D).

These three steps ensure that the harm domain is comprehensively evaluated within the CM method, fulfilling the Directive’s HBA requirement (see Figure 3). The project application must fully explain these aspects so that the relevant committee can incorporate them into their deliberations when evaluating this domain. Figure 4 provides a classification of anticipated harm, categorizing harmful procedures using an escalation model to simplify decision-making. The escalation model provides visual guidance illustrating how severity gradually increases in duration and intensity, which is reflected in an increase in the severity category. Transitioning from one category to the next may not be clear-cut in certain cases; consequently, a gradual transition is indicated to provide the necessary flexibility. The escalation model provides a tool for both the project applicant and the committee members to determine the severity and highlight overlapping areas. To demonstrate the practical application of the escalation model, the illustrative cancer study described previously (see Table 5) has been integrated into Figure 4. This provides a clearer illustration of the severity classification for each of the study groups. A project’s harm domain can then be categorized using the four severity categories introduced in Table 5 (i.e., non-recovery, mild, moderate, and severe).

### 3.3 Benefit domain: creating a real-world impact

The (terminological) differentiation between outcome and benefit is a critical gap in current HBA methods and most GDs (2, 24). To our knowledge, this is the first study that makes a clear distinction between those two inherently different aspects in an HBA methodology. The benefit domain represents the promise dimension of a project (6). A project’s benefit in providing a positive real-world impact can be determined only by evaluating the likelihood of achieving the benefit and whether it will have a net positive effect on humans, animals, and/or the environment. However, this is not straightforward and risks inappropriate speculation (24). The available GDs have attempted to categorize project benefits into direct and indirect benefits. As described in the British GD (28), direct benefits can have an immediate impact at the project’s conclusion, are attainable and/or quantifiable, and are directly attributable to the project (e.g., validation of a developed diagnostic test to detect a specific disease). Indirect benefits have a wider scope and can result in a project’s broader impact on other research areas, creating benefits that may not be obvious and/or realizable immediately or linked directly to the project.

The British and Belgian GDs provided components that can be applied to the CM approach. The British GD includes specific questions to facilitate domain characterization. In general, determining a project’s benefits involves identifying and describing its benefits, its beneficiaries, how they will benefit, and when the benefit will be achieved. Within the CM, assessing the benefit domain requires identifying the benefit streams, which assigns the anticipated benefit across the following relevant areas (as summarized in the Belgian GD): social, socioeconomic, scientific, educational, and safety/efficacy testing. The project applicant must highlight the project’s relevance to one or more of these benefit streams; for example, a project focused on basic research may impact the social, scientific, and educational streams. As recommended in the British GD, this can be facilitated by requiring that the applicant answer specific project-related questions. By using these streams, the applicant organizes the expected benefit into specific areas, providing the reviewing committee with a clearer understanding of where a real-world impact can be expected.

**Figure 5:**
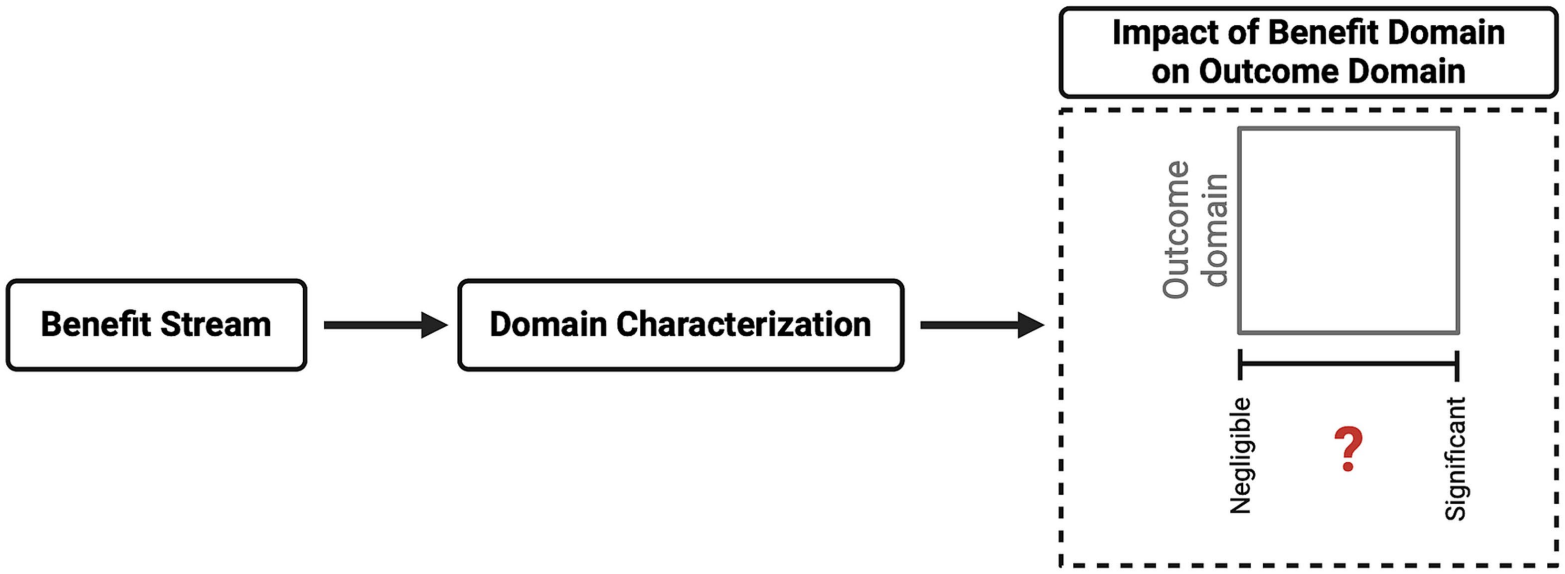
Benefit domain evaluation.

A clearer understanding of a benefit’s impact on certain areas will enable a reviewing committee to recognize its core influence. The committee must then deliberate on the information presented and determine the likelihood that the project will produce the expected benefit. The CM approach recommends that the committee categorizes a project’s final benefit into one of four categories: negligible, low, significant, or substantial (see Figure 5).

It must be acknowledged that the benefit domain evaluation is not a universal approach. This is a result of several factors, including the broad variability of research and associated goals, individual national relevance of or constraints on specific research areas, and the challenge of predicting actual net benefits. Nevertheless, the committee must make an informed decision regarding the promise domain and the likelihood of it being realized.

This potential vagueness may mean that this step in the CM method provides only limited value—currently, this may be the case. However, similar to the harm domain, explicitly addressing this aspect broadens the benefit domain by generating further examples and a knowledge base that can be used in new situations. Therefore, given that this step has not been explicitly included previously, documenting the reasons for and against particular categorizations can be immensely valuable, enabling reviewing committees to learn from both their own past decisions and those of other committees.

### 3.4 Justification domain: providing reasons, not numbers

Thus far, balancing harm and benefit forms the core of the HBA. The MM and DM approaches diverge methodologically in this respect, with the MM method using a strict metric, and the DM method providing flexibility through deliberation but at the risk of inconsistency; this makes it challenging to draw comparisons when justifying decisions. The Swiss GD is the only GD that includes a balancing scheme that outlines when a project’s harm is justified (i.e., when the harm or benefit domain is outweighed or outscored) (31, 32). However, the practical applicability of this approach and its overall operational consistency remain uncertain.

**Figure 6:**
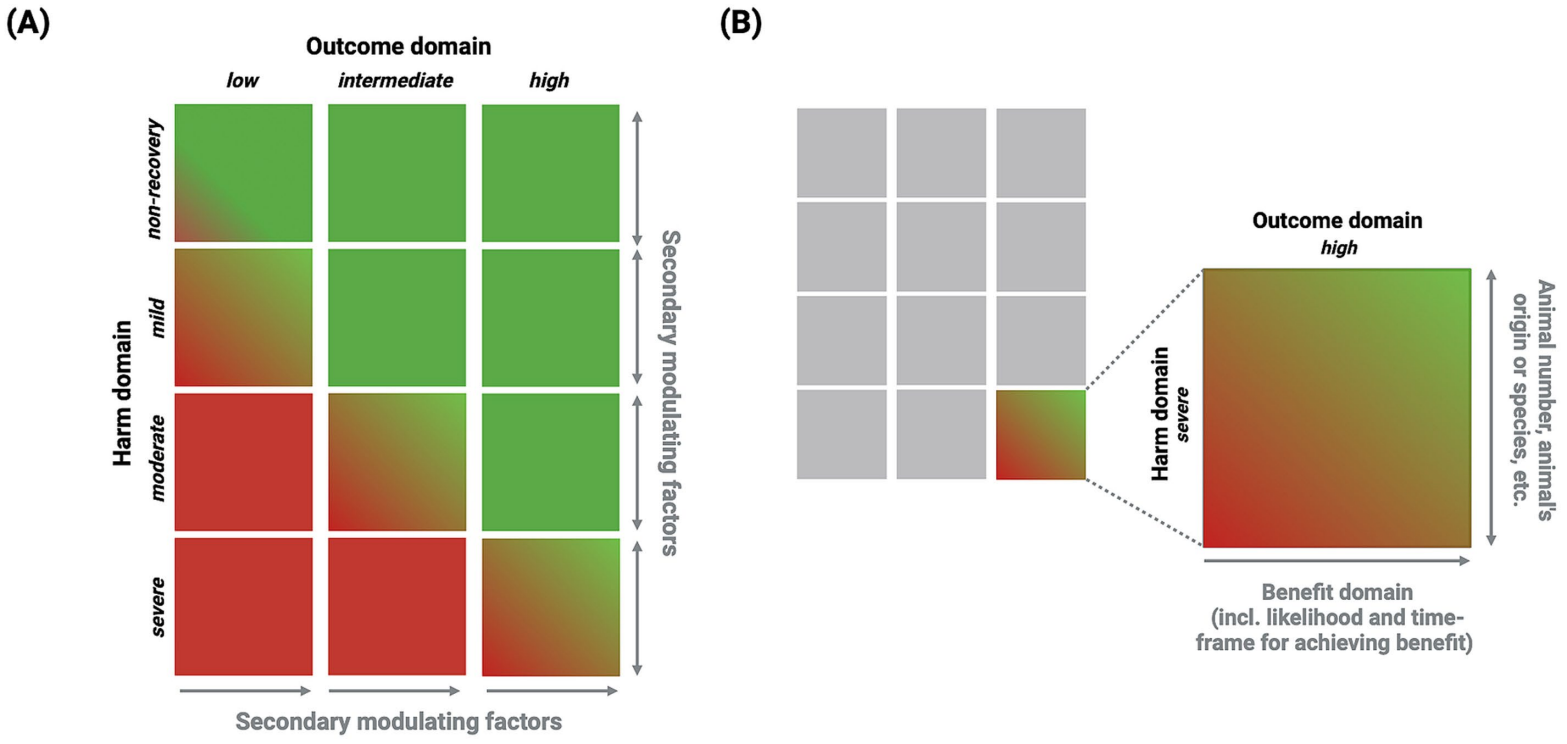
Justification domain. **(A)** Decision matrix: The outcome domain is represented by three categories (low, intermediate, and high) in a similar way to the harm domain (non-recovery, mild, moderate, and severe). Secondary MFs act as modulators for an individual harm–outcome relationship (i.e., a specific matrix element). The red color indicates that the harm domain outweighs the outcome domain, signifying disapproval of the project, whereas the green color signifies that the outcome domain either outweighs or is in balance with the harm domain, denoting approval of the project. **(B)** Matrix element: This shows a single matrix element and illustrates how the relationship between harm and outcome is affected, within limits, by secondary MFs and the benefit domain, respectively (see Table 4 for a comprehensive list of the secondary MFs influencing the harm domain).

The CM method introduces a new approach for the justification domain by placing the harm and outcome domains (not the benefit domain) in a decision matrix that directly relates these domains to each other (see Figure 6A). This relationship is further nuanced using MFs as an additional assessment layer.

Primary MFs directly affect an individual animal’s experience and have an immediate effect. Therefore, these MFs need to be considered when determining a project’s harm domain category. In contrast, secondary MFs have a more indirect effect on the animal’s harm experience, modifying the individual harm–outcome relationship of a project by alleviating or exacerbating harm.

Similarly, the likelihood of achieving a project’s anticipated benefit can influence the harm–outcome relationship and, more explicitly, the outcome domain. As such, the benefit domain acts as an MF for the outcome domain. The actual anticipated benefit, its beneficiaries, areas of impact, and the likelihood of achieving the benefit (i.e., when a positive real-world impact can be expected) have roles in modulating the outcome domain.

To our knowledge, our study represents the first time within an HBA framework that the harm domain is evaluated relative to the outcome domain rather than the benefit domain. This harm–outcome approach establishes a direct connection between the harm to animals and a project’s scientific outcome, thereby relating causally linked aspects rather than vaguely associated dimensions. This reorientation aligns HBA logic with the idea that foreseeable outcomes (which are prerequisites for project benefits) are inherently connected to foreseeable harms; the identified harm is a direct consequence of reaching a particular outcome and cannot be alleviated by the 3Rs prerequisite.

By recognizing that outcome is a fundamental domain, the CM method shows that benefit represents a potential achievement that is dependent on the successful realization of the outcome specified by a project and various other factors. The proposed decision matrix visually illustrates how the relevant domains interact with each other and are further influenced by MFs (see Figure 6). In this scenario, harm infliction represents an unintended yet foreseeable and necessary means to achieve a project’s scientific goal—namely, the outcome. To reach this outcome, planned and foreseeable harm is an unavoidable disadvantage; however, such harm must be minimized as much as possible by adhering to the provisions of the 3Rs as a prerequisite. If such harms are not effectively managed by the 3Rs, the project is deemed unacceptable and illegal (see also the previous discussion in Section 3.2 on the number of animals being either correct or incorrect rather than high or low).

As such, it is also reasonable to argue for justifying harms based on the project outcome rather than its benefit. This is because the outcome is directly linked to unavoidable harm, while the benefit is not. The following example illustrates this further: a project aims to test the efficacy of a substance for treating a specific disease. Determining whether the substance is effective is the project’s outcome, which may require a certain, unavoidable number of animals to be harmed. Since this number must be adequate and is either correct or incorrect, the outcome cannot be achieved with fewer animals—if there is one animal less than needed, the outcome will not be reached, and if there is one animal too many, the project will be considered illegal. Thus, reducing harm beyond the 3Rs requirement directly affects the achievement of the outcome. The *harm investment* is the unavoidable consequence of reaching the desired outcome.

In the benefit domain, this relationship is much looser. Whether future benefits arise from the knowledge gained (i.e., the outcome) is a separate issue and does not become more or less likely owing to an increase or decrease in the harm inflicted on animals. An increase in the benefit does not require more harm, nor does a decrease in the number of animals compromise future benefits; rather, they simply jeopardize the outcome and the legality of reaching it. Therefore, the direct relationship between outcome and harm, rather than between benefit and harm, provides the basis for a satisfactory HBA. Consequently, the justification relationship should focus on harm–outcome rather than harm–benefit, relegating the benefit to a secondary modulating role.

Hence, secondary MFs are considered additional factors to adjust the harm–outcome relationship. Within the decision matrix (see Figure 6B), secondary MFs can influence the harm domain by increasing or reducing the overall harm experienced by an animal. In contrast, the benefit domain gradually influences the outcome relationship. The benefit domain progresses along a continuous vector that can shift the outcome domain within certain limits. This is particularly relevant to the non-recovery category of the harm domain, where secondary MFs have a role in evaluating non-recovery projects with a low outcome domain.

Moreover, the decision matrix relies on the justifications provided by the project applicant for the previous domain evaluations. Effective decision-making using the matrix requires reasoning that substantiates the corresponding domain categorizations. While a metric approach may be implied by a matrix, it is important to recognize that this is a dynamic model that is dependent on satisfactory justifications from the project applicant, and as an evolving model, it builds on scenarios that shape its future application.

In summary, Figure 6A shows the decision matrix for the justification domain, while Figure 6B depicts the matrix for a single element. The decision matrix can guide a reviewing committee in identifying potential weaknesses in a project application, such as when the harm domain significantly outweighs the outcome domain or where a project is unlikely to generate a benefit. It also highlights the interaction between the harm and outcome domains, as well as their modulators, such as the influence of the harm domain’s secondary MFs and the benefit domain on the outcome domain.

### 3.5 Ethical considerations domain: ethics within legal limits

The Directive includes “taking into account ethical considerations” as one of the HBA domains (1). However, this domain is either particularly vague or is not considered at all in the available GDs. Most of the GDs, including the European Commission’s GD as the overarching framework, do not define this domain; furthermore, there are no recommendations regarding its implementation. Only the Dutch GD includes a recommendation, stating that reviewers should consider ethical considerations when evaluating harms and benefits (33); however, it provides no guidance on operational elements. Instead, it recommends reflections based on ethical theories, such as virtue ethics, consequentialism, and deontological ethics.

This well-intentioned proposal presents a significant risk for reviewing committees; if ethical considerations extend beyond or fall short of what is legally required by the Directive, decisions may be made without a legal foundation. Authorities must make decisions following the principle of legality and, thus, have a legal basis (this may overlap with ethics but is not the same); if not, the principle of legality is infringed, and the reviewing committee may violate the rule of law. As such, we argue that incorporating ethical considerations into HBA is affected by a confusion between the roles of ethics and law in a democratic society.

It is evident that HBA reflects an ethical position, which is—in general terms—consequentialist (the net positive and negative consequences defines the quality of a project), pathocentric (the Directive mainly focuses on the negative subjective experience of animals, such as pain, suffering, and distress), and hierarchic (not all animals treated equally for different reasons; e.g., human animals, animals that must not be used, animals that are not considered animals according to the Directive such as insects or embryos in early developmental stages). There is nothing wrong with considering such ethical positions if they are based on existing law, as this gives them a legal basis. However, ethical aspects and positions can be considered in an HBA carried out by a legal authority only if they have a basis in law or relevant enforced legal requirements. If an ethical requirement exceeds the legal requirements (e.g., in the case of abolitionism) or falls short (e.g., in the case of particular forms of contractarianism), it must not guide an authority’s decision—owing to the rule of law principle—whether this is via an advising committee or otherwise.

To illustrate this, contractarianism and the animal rights view represent two ethical positions. Most contractarians argue that only beings with the ability to consent and bind themselves to the decisions they make are members of the moral community and have moral status (34–36). As such, only beings able to act in accordance with rights and duties (i.e., full moral agents) are morally considerable. As a consequence, animals used in animal research that are legally protected would not be under moral protection due to the fact that they are not full moral agents. Applying a contractarian view would make HBA a very quick endeavor because most, if not all, non-human animals would not count (only those that are moral agents); however, this would be illegal because harm to animals (as defined by the Directive) has to be considered in an HBA. In summary, as long as an authority’s decisions are made based on the rule of law principle, ethical claims beyond existing law must not be considered.

In contrast to contractarianism, the animal rights view (37–39) considers that animals with certain abilities (e.g., sentience) must not be instrumentalized. Hence, an HBA would again not be necessary from an ethical perspective because HBA is a tool that considers whether a certain level of instrumentalization is justified. Applying a strict animal rights view, this would not be an option since instrumentalizations must not occur at all, regardless of the outcome (or the benefit). Again, employing this position for an HBA has no legal substantiation—quite the opposite, in fact, as the Directive allows animal research (gaining knowledge and benefits by instrumentalizing animals) under certain conditions. Taking such ethical considerations into account would again infringe the rule of law principle.

These two examples illustrate that “taking ethical considerations into account” cannot influence the evaluation to exceed or fail to meet actual legal requirements (36). Hence, it could be argued that countries that avoid implementing this domain are more secure since only legal requirements can have a role in a reviewing committee’s recommendation—otherwise, the rule of law may be violated. Of course, there may be flexibility, but only within the given legal limits. As such, ethical considerations must remain within the legal framework.

This raises the question of why such a provision was included in the Directive and why it has not been subsequently deleted. As our argument shows, we believe that it would have been preferable not to integrate this ethical domain into HBA to avoid the risk of decision-making that infringes actual laws or the rule of law principle. Accordingly, it is surprising that the Dutch GD refers to ethical approaches such as virtue ethics, deontology, and consequentialism—by way of an example, virtue ethics focuses not on the quality of an action but on the character of its agent; is it a good idea to evaluate a research proposal based on the virtuous character of the researcher, applicant, or nurse involved? As such, it is doubtful whether the Dutch GD has taken its own recommendation seriously as it risks violating the rule of law principle. If it is not serious, then why was it included in the first place? Fortunately, only one of the countries examined by Hajosi and Grimm (2) had implemented this domain.

In summary, we believe that the ethical considerations domain can form part of an HBA only if this ethical component is substantiated in the legislation of the relevant Member States. This is the same as saying that only legal requirements can have a role when evaluating project proposals. As we have integrated all of the other HBA domains when developing the CM method, we believe that integrating further aspects is neither necessary nor feasible. As such, all relevant ethical considerations must be explicit in or intended by law if they are to be considered. If they are not, then they should not influence the decisions of the reviewing committee or the competent authority.

As a result, we have removed this problematic domain from the CM approach as it is implicit in HBA and cannot add to an HBA’s legal requirements. The notion of incorporating ethical considerations into a legal procedure stems from a confusion between ethics and law that can be clarified by referring to the rule of law principle.

### 3.6 The compound model as a practical harm–benefit analysis tool: guidance for project applicants and reviewing committees

The comprehensive HBA method described in the preceding sections is included as an addendum to this study (see Appendix 1). This document should be used when a project undergoes an HBA. It is designed to support the work of the applicant, authority, and committee members by applying a transparent methodology that aligns with the Directive’s framework.

## 4 Discussion

The inherent challenges of the current HBA methods—the MM and DM approaches—provided the starting point (2, 3) for this study. However, how to practically navigate between these two models and develop a transparent composite method needed to be addressed. While the two models agreed that the harm inflicted on animals used for scientific purposes requires a justification based on a project’s potential to achieve a positive impact, how this should be done was unclear. To overcome this situation, this study analyzed the GDs from six European countries, as well as the EU’s overarching framework, to produce a composite CM method for conducting HBA (see Appendix 1).

### 4.1 Putting the puzzle together: do the pieces fit?

The results indicate that a CM method can be constructed based on the identified materials that both integrates all of the relevant HBA domains and shifts the focus of several of them. The first step in this method involves evaluating the outcome domain through a scientific evaluation. This is the first step in an HBA—conducted before the other domains are analyzed—because the project outcome has a foundational role for all subsequent stages. The scientific evaluation of the outcome domain can be undertaken through a peer-review process of a funding agency or a reviewing committee’s evaluation. Given that a committee often includes members from diverse (non)scientific backgrounds, subject matter experts should be consulted to ensure that scientific rigor is reliably assessed. The implication of this dual method (funding agency versus external subject matter experts) on an HBA is that a committee is not responsible for determining whether a project can generate the predicted outcome; rather, its focus is shifted toward forming an opinion on the relevance of the outcome and on ensuring that the outcome is within the legal framework by assigning it to an outcome stream—which reflect the Directive’s specified research purposes that animals can be used for—and further characterizing the outcome. The multidisciplinary backgrounds of the committee members and the importance of certain research areas for some committees can influence a committee’s decision on relevance. These influences may be inevitable and mean that the evaluation may be subject to bias. However, this approach emphasizes the importance of the outcome domain and provides a standardized evaluation of this foundational element.

The subsequent harm domain evaluation is widely regarded as the most mature HBA domain owing to its regulatory clarity—namely, the Directive’s severity categories and its Annex VIII provisions (1). Our CM method redefines the longstanding but risky relationship between harm and benefit and instead links harm to the outcome domain. As such, harm is necessary to reach the anticipated scientific outcome rather than the anticipated benefit (with ramifications discussed shortly). This disentanglement of harm and benefit makes it clear that the causal relationship between harm and outcome prevails if the 3Rs are implemented appropriately.

Given the relatively clear indicators and features of the harm domain, the CM method refines the current approach. As such, harms are considered using an escalation model that facilitates decision-making on the severity of individual procedures and supports the identification of potential overlapping areas. This visual, hierarchical guidance can be used to determine whether procedures are scaled correctly and whether an escalation of harm should be scrutinized more extensively.

Secondary MFs can affect the final harm domain categorization based on their impact on an animal. The European Commission’s and Belgian GDs provided a comprehensive list of MFs (27, 29). However, these MFs need to be differentiated into primary and secondary MFs that either directly or indirectly influence an animal’s experience of harm (see Table 4). As discussed in Section 3, the CM method does not consider that the total number of animals is a significant MF that can influence a project’s severity category. The notion of including animal numbers is further discussed in Section 4.2.

The harm domain’s structural clarity provides a positive example for refining the remaining HBA domains, which exhibit obvious gaps. The benefit domain, as the promise dimension, which is focused on (future) positive impacts in real-world settings, is included in the CM method as an additional layer of consideration—a MF—for the outcome domain. Benefits are defined, characterized, and assigned to streams in the benefit domain evaluation, creating a more nuanced view of the impacted areas. Considering these streams critically, categorizing benefits into only five streams may appear too stringent and unduly flexible. However, these stream categories would provide clarity for reviewing committees on the areas that would be affected. Given that evaluating the likelihood of a benefit always includes a degree of uncertainty, the CM method does not use this evaluation to determine whether harm is justified; only the outcome domain is used in this justification.

The justification domain is also changed in the CM method. The evaluation of harm and—now—outcome is performed using a (4 × 3) decision matrix that assesses the relationship of these domains to each other. Assessing domains using a decision matrix as a table or three-dimensional design is not new. Relevant HBA accounts using such formats have been described in existing literature (6, 26, 40). However, in contrast to the Swiss GD (31, 32), for example, the CM decision matrix avoids prioritizing human interests and instead focuses on the direct relationship between harm and outcome, as we believe that these domains should be considered equally important.

The benefit domain and secondary MFs have an important role within the individual matrix elements (see Figure 6B). The example included in Figure 6B shows that the individual harm–outcome relationship is placed in an enclosed setting with boundaries that must be respected by the benefit domain and secondary MFs. This is a critical aspect of the CM approach: the influence of the benefit domain—or promise dimension—on the harm–outcome relationship is limited to a defined area. This creates a form of standardization for reviewing committees but also maintains the necessary flexibility by considering secondary MFs.

In conclusion, the CM method comprises four primary domains: outcome, harm, benefit, and justification. Only these four domains, when considered together, complete the HBA puzzle.

### 4.2 Addressing the elephants in the room: removing the role of ethics and reshaping the influence of the total number of animals

The CM method entirely forgoes the ethical considerations domain. As described in Section 3.5, the ethics domain has been under scrutiny ever since its inclusion in the Directive’s HBA requirement. Removing the ethics domain from the CM method may remedy some of the weaknesses of HBA, including a lack of transparency, coherence, feasibility, and comprehensibility. The ethics domain does not fit as a piece of the HBA puzzle; consequently, it is not included in the CM as a separate domain. Rather, it has been argued that the ethics domain is implicit in the HBA process itself (2, 6), with several normative dimensions integrated into the legal requirements that act as its basis.

Another notable exclusion from the CM method is that the total number of animals MF does not have a primary role in the model. The systemic reasoning for this is that by differentiating between the outcome and benefit domains, it becomes clear that the outcome cannot be achieved by using fewer animals than originally planned. That is to say, if the 3Rs requirement is satisfied before conducting the HBA, this will ensure that the harm animals experience to achieve the scientific outcome is minimized. This means that the outcome can only be achieved by using a certain number of animals, regardless of what that number actually is. The project cannot reduce this number because it would not be legal as the project could not then ensure that the scientific outcome is generated. Thus, the requirement is not seeking a minimum number of animals but a minimum that cannot be lowered any further. For this reason, the total number of animals should not be included as a key aspect. Furthermore, this approach avoids the need to consider a threshold for a “high” number—or upper limit—of animals (e.g., whether 10 or 10,000 animals, regardless of the species, is too many) and focuses on the requirement to protect the individual by avoiding the summarization of harm across individuals.

The Directive is clear that a project’s severity category is “based on the most severe effects likely to be experienced by an individual animal” (1). This indicates that the legal basis for the severity category is the individual animal that is most severely affected rather than the total number of animals or the relative number of animals in different severity categories. Consequently, the number of animals is not a primary MF that would ameliorate (or worsen) the severity experienced by the most severely affected animal, even after applying all refinement options.

This notion applies to both ends of the harm gradient: projects that cause severe harm but use a small number of animals will be considered as having greater harm than projects that cause moderate harm but use a larger number of animals. This reflects the idea of prioritizing the harm experienced by individual animals, which is replicated in the severity categories. However, representing the proportions of the different severity categories and the relative numbers of animals in each category does provide a clearer picture of a project, even though this does not alter the overall categorization.

By excluding the total number of animals as a decisive factor, the CM method risks resistance from stakeholders since committee members often use the total number of animals as a measure of the overall harm, which then shapes their decisions on the harm domain evaluation. However, in accordance with the Directive, we argue that this total number does not influence the severity classification. Nevertheless, the allocation of animals to individual severity categories does help us to understand the overall impact on the animals. The distribution of harm mirrored in the severity categories across animals’ used addresses the intuition that an experiment harming more animals is worse than harming less without questioning the severity category as the main criteria to classify harm according to the Directive. As such, this is included in the CM as a secondary factor.

### 4.3 Reviewer’s discretion advised: laying the path for reasoned judgment

Removing the ethics domain from HBA may appear to be a retrograde step for animal protection—deprioritizing animal welfare by removing the ethical debate. Ethical considerations should provide a framework of moral acceptance for using animals in research, but it should not dictate an authority’s decision that must be based on enacted law in the review process. As such, retaining the ethics domain in the CM method would create a weaker and problematic framework, making the reviewing committee’s decisions susceptible to criticism. The committee’s work must produce a *reasoned discretionary judgment* that is robust, can withstand critique or appeal, and remains within the committee’s discretion as stipulated in law. The distinction between the respective functional areas of ethics and the law within liberal societies must be respected, which is itself an ethical goal.

The CM prioritizes legal compliance over ethical reflection, which could make it less appealing to committee members who believe that ethics should be an essential component of HBA. While this may create the impression that ethical deliberations have been sidelined, we argue that HBA is an ethical tool by implication and that only aspects mandated by law can and should be included in an HBA.

Furthermore, by making a distinction between ethics and law, ethics—as moral philosophy—achieves one of its noble goals by reflecting on current law. If current law can be critically reviewed from an ethical perspective, then this should be done. Clearly, this is to say that the legal framework can be changed based on ethical reflection and democratic procedures. However, as long as the legal framework is in power, the review process has to be carried out within it. Ethical positions can guide the decisions of authorities only if they have received a majority in a law-giving procedure.

### 4.4 The compound model for harm–benefit analysis: a pragmatic solution to the current challenges involves limitations

This study describes a novel methodological approach to HBA during the project review process by combining elements from available guidance. Owing to the gaps in the available GDs, we were restricted in our attempt to create a broad, comprehensive methodology; this led us to develop pragmatic gap fillers. As the CM method is assembled by integrating elements from various GDs, there is a risk of incoherent implementation by different reviewing authorities or Member States.

In addition, the practical feasibility and accountability of this new method remains to be determined and will be subject to scrutiny because HBA accounts—regardless of their form—are dynamic and are expanded and defined through application. This methodological validation is a key limitation that should be addressed through empirical studies investigating its implementation by reviewing authorities.

A future goal is to enhance and expand the CM’s adaptation by defining clearer examples of the benefit domain’s impact areas to simplify categorization. Given the speculative characteristics of benefit evaluation (promise dimension), its assessment remains challenging, even within the CM framework. One approach to address the uncertainty associated with the benefit domain would be to produce a framework similar to that for the harm domain in Annex VIII. As such, an analogous model, like the escalation model (see Figure 4), could be developed.

Furthermore, implementing the justification domain and operationalizing the decision matrix needs to be validated; this could be done by surveying committees and authorities to assess their application. As such, the matrix will evolve through experience and should not be regarded—at this point—as a fixed solution. The decision matrix’s effectiveness depends on how reviewing bodies interpret and operationalize it in their decision-making processes. Biases, conflicting and contextual priorities, and institutional pressures can jeopardize the matrix’s objectivity. Making the review process transparent is crucial, enhancing trust in committee decisions and their (societal) acceptance (41).

Validation of the CM would benefit from input from multiple committees. Future studies could also investigate the implementation of the proposed changes in the CM, particularly the realigned relationship between outcome and harm, the differentiation between outcome and benefit, the inclusion of secondary MFs, the operationalization of the decision matrix, and the removal of the ethics account and the total number of animals as a significant modulator.

While there are questions regarding the CM method’s adaptation, it represents a pragmatic approach that addresses the current gaps and pitfalls of HBA. Given that HBA has seemingly been stuck in a draft mode, we have sought to create a composite methodology that merges significant parts of current HBA methodologies, concepts, and available GDs that have remained somewhat fragmentary to this point. As such, the CM is a practical tool (see Appendix 1) with transparent guidance that accords with the Directive. We expect that this new method will find critics as well as supporters; this will further debate and help to solve the practical challenges of HBA. Whether the CM method can address the current HBA challenges by effectively piecing together incomplete HBA elements remains to be seen; however, this study provides a foundation for a promising way forward.

## Data Availability

The original contributions presented in the study are included in the article/Supplementary material, further inquiries can be directed to the corresponding author.
