# Supplementary material for: Solving the riddle by puzzling it out: overcoming the challenges of harm–benefit analysis by assembling a compound model and practice-oriented tool
Source: Front Vet Sci. 2025 Dec 11;12:1593954. doi: 10.3389/fvets.2025.1593954 (PMC12739955; doi:10.3389/fvets.2025.1593954)
Supplement: Supplementary file 1 [file Supplementary_file_1.zip › Supplementary Material/Appendix 1.PDF]

Harm-Benefit Analysis – Compound Model

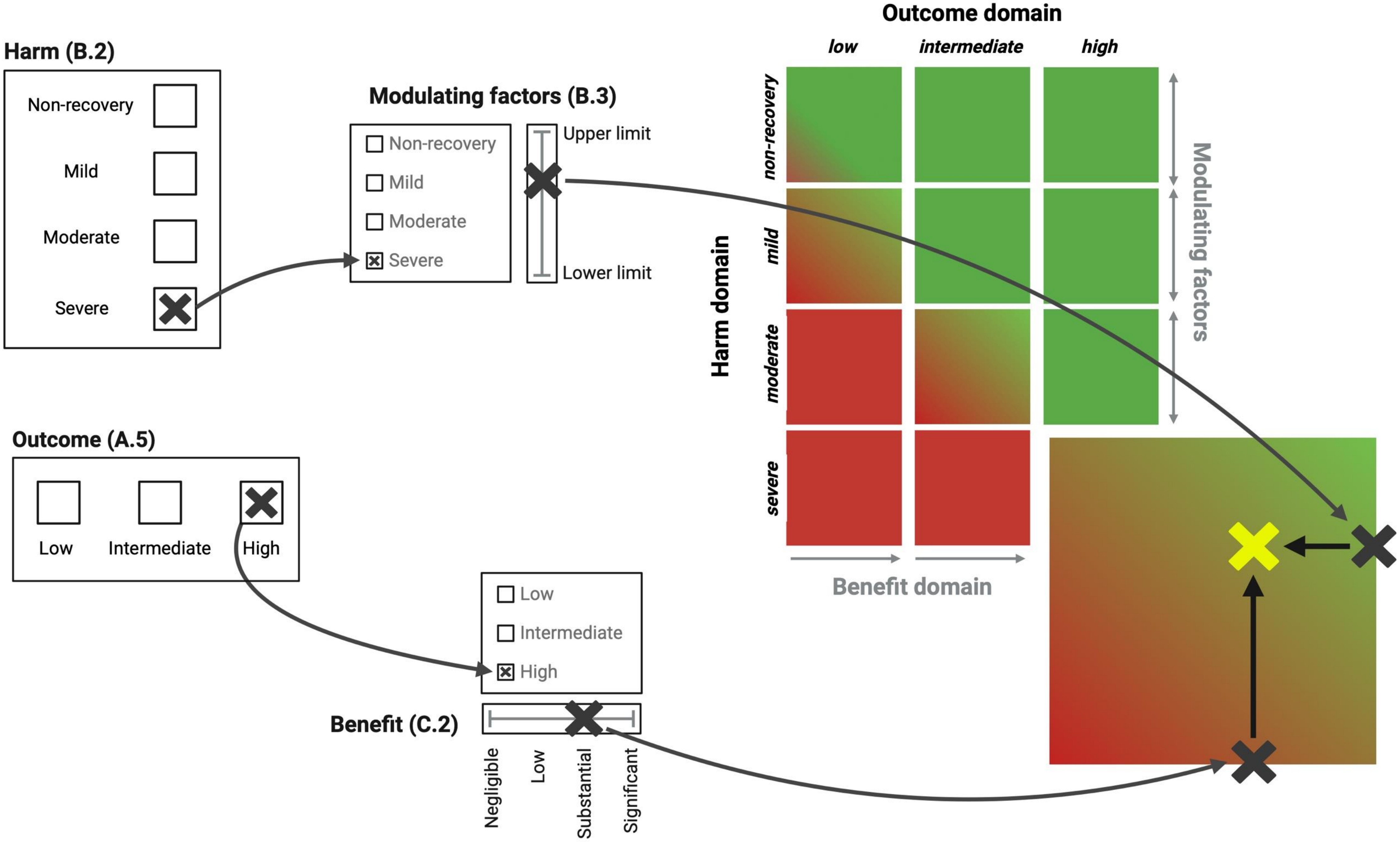

A: Outcome domain – Nature and Quality Assurance of Knowledge Gain (A.1-A.3)

A.1 Description of anticipated knowledge

Project applicant	Committee member
Please briefly state the project's anticipated knowledge gain, i.e., outcome, by stating it below or referring to the relevant section of the project proposal:	Has the project applicant clearly stated the project's outcome?<br><br>❑ Yes<br>❑ No

A.2 Assignment of project to outcome streams

Please assign the project's outcome to at least one of the following outcome streams (Article 5, EU Directive 2010/63/EU).<br><br>*Note:* If a project does not fall within at least one outcome stream, it does not align with the relevant purposes described in Article 5 of EU Directive 2010/63/EU.	❑ Basic research<br>❑ Translational/applied research<br>❑ Protection of natural environment<br>❑ Higher education or training<br>❑ Forensic inquiries<br>❑ Quality/efficacy/safety testing<br>❑ Preservation of the species<br>❑ Not applicable	❑ Basic research<br>❑ Translational/applied research<br>❑ Protection of natural environment<br>❑ Higher education or training<br>❑ Forensic inquiries<br>❑ Quality/efficacy/safety testing<br>❑ Preservation of the species<br>❑ Not applicable

A.3 Quality assurance of the project

Has a sufficient scientific review been performed for the project?	❑ Yes<br>❑ No<br><br>If "Yes", describe briefly the review procedure:	❑ Yes<br>❑ No

A: Outcome domain – Characterizing the Outcome Domain (A.4)

		Project applicant	Committee member
		To substantiate your selection, please refer to the relevant section in the project proposal:	
**A.4.1**	To what extent does the project address relevant scientific challenges?	❑ Low<br>❑ Intermediate<br>❑ High<br>❑ Not applicable	❑ Low<br>❑ Intermediate<br>❑ High<br>❑ Not applicable
**A.4.2**	To what extent is the proposed scientific approach feasible for addressing the problem in question?	❑ Low<br>❑ Intermediate<br>❑ High<br>❑ Not applicable	❑ Low<br>❑ Intermediate<br>❑ High<br>❑ Not applicable
**A.4.3**	To what extent is the described methodology robust and sound for addressing the scientific question and yielding the anticipated outcome?	❑ Low<br>❑ Intermediate<br>❑ High<br>❑ Not applicable	❑ Low<br>❑ Intermediate<br>❑ High<br>❑ Not applicable
**A.4.4**	To what extent is the proposed timeline appropriate and realistic for generating the anticipated outcome?	❑ Low<br>❑ Intermediate<br>❑ High<br>❑ Not applicable	❑ Low<br>❑ Intermediate<br>❑ High<br>❑ Not applicable
**A.4.5**	To what extent are the resources (incl. personnel and respective time commitments) adequate for the planned project proposal?	❑ Low<br>❑ Intermediate<br>❑ High<br>❑ Not applicable	❑ Low<br>❑ Intermediate<br>❑ High<br>❑ Not applicable
**A.4.6**	To what extent is the expertise of the personnel (incl. the principal investigator) sufficient to work with animals and carry out the project to achieve the anticipated outcome?	❑ Low<br>❑ Intermediate<br>❑ High<br>❑ Not applicable	❑ Low<br>❑ Intermediate<br>❑ High<br>❑ Not applicable

A: Outcome domain – Selecting the Outcome Category (A.5)

Please select the adequate category for the domain evaluation by placing an "X" in the corresponding box, based on the average of the previously selected answers in A.4.1-A.4.6.

Note: The provided answer will feed into the decision matrix in the end.

Project applicant			Committee member		
☐	☐	☐	☐	☐	☐
Low	Intermediate	High	Low	Intermediate	High

B: Harm domain – Severity Classification for all Study Groups (B.1). *Note*: Please provide one page for each study group.

Study group 1

Number of animals:

Please classify the severity of all procedures for study group 1.

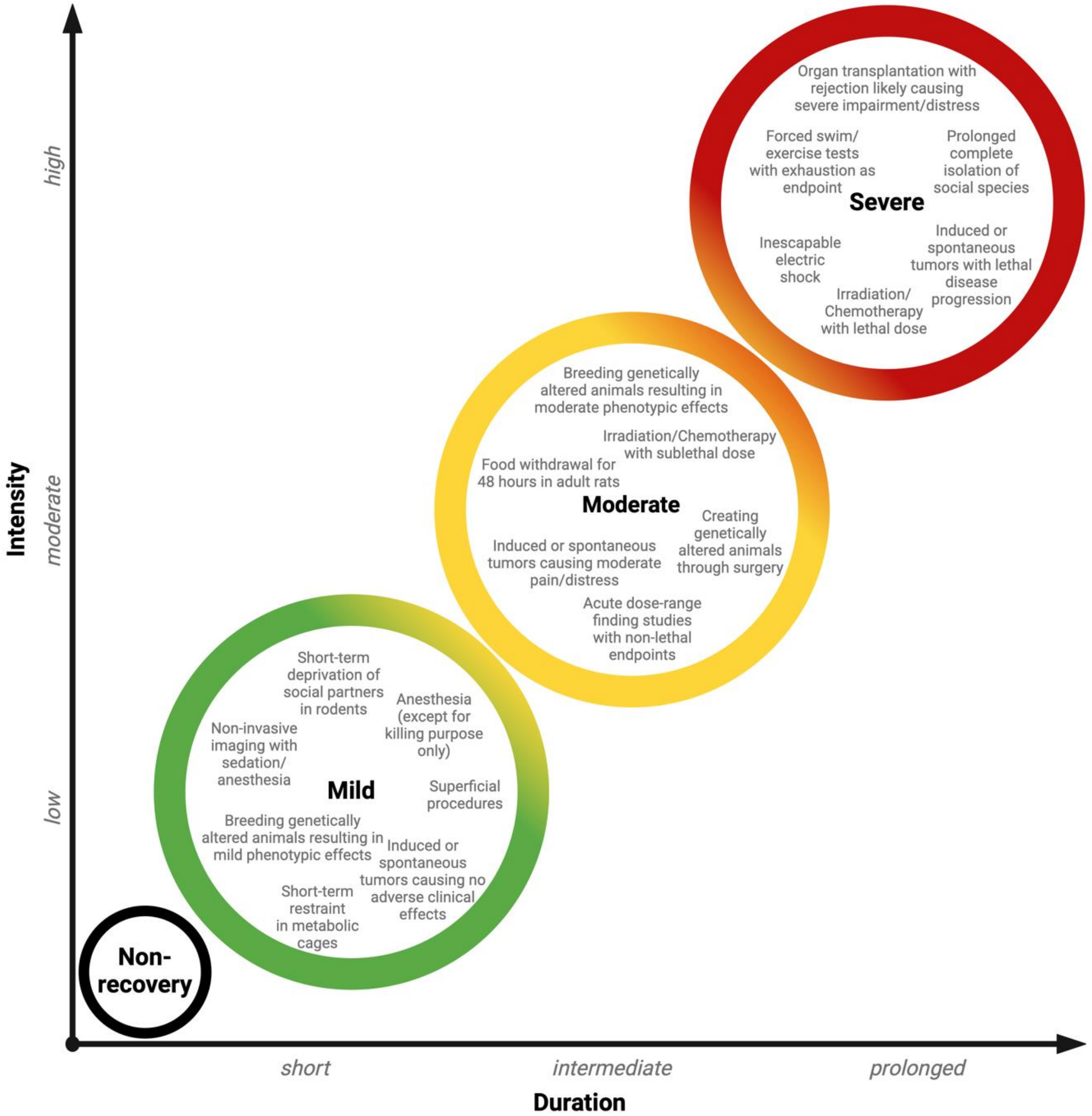

Project applicant

Instructions: Identify all harms linked to the procedures, assign each procedure with a corresponding number in the escalation model, place a bubble with a number within the escalation model, and list them accordingly.

List of all procedures for study group 1:

(1) ______________________________

(2) ______________________________

(3) ______________________________

(4) ______________________________

(etc.) ______________________________

Please classify the severity for this study group by placing an "X" in the corresponding box below:

☐ Non-recovery ☐ Mild ☐ Moderate ☐ Severe

Committee member

Are the relevant harms adequately classified in the escalation model?

❑ Yes
❑ No

Has the severity of the study group been classified adequately?

❑ Yes
❑ No

B: Harm domain – Severity Classification for all Study Groups (B.1). *Note*: Please provide one page for each study group.

Study group 2

Number of animals:

Please classify the severity of all procedures for study group 2.

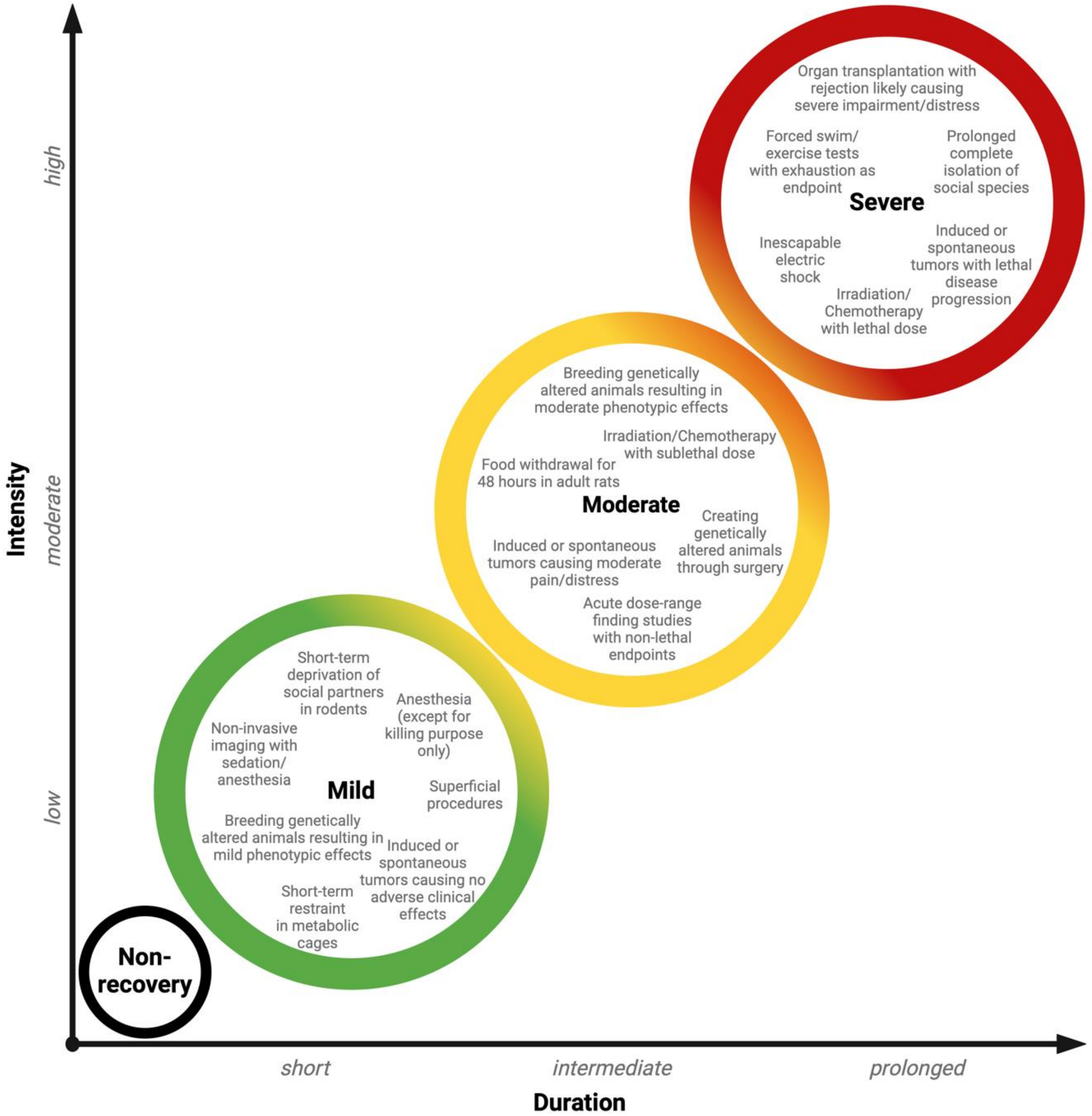

Project applicant

Instructions: Identify all harms linked to the procedures, assign each procedure with a corresponding number in the escalation model, place a bubble with a number within the escalation model, and list them accordingly.

List of all procedures for study group 2:

1 ______________________

2 ______________________

3 ______________________

4 ______________________

etc. ______________________

Please classify the severity for this study group by placing an "X" in the corresponding box below:

☐ Non-recovery ☐ Mild ☐ Moderate ☐ Severe

Committee member

Are the relevant harms adequately classified in the escalation model?

❑ Yes
❑ No

Has the severity of the study group been classified adequately?

❑ Yes
❑ No

B: Harm domain – Severity Classification for all Study Groups (B.1). *Note*: Please provide one page for each study group.

Study group X

Number of animals:

Please classify the severity of all procedures for study group X.

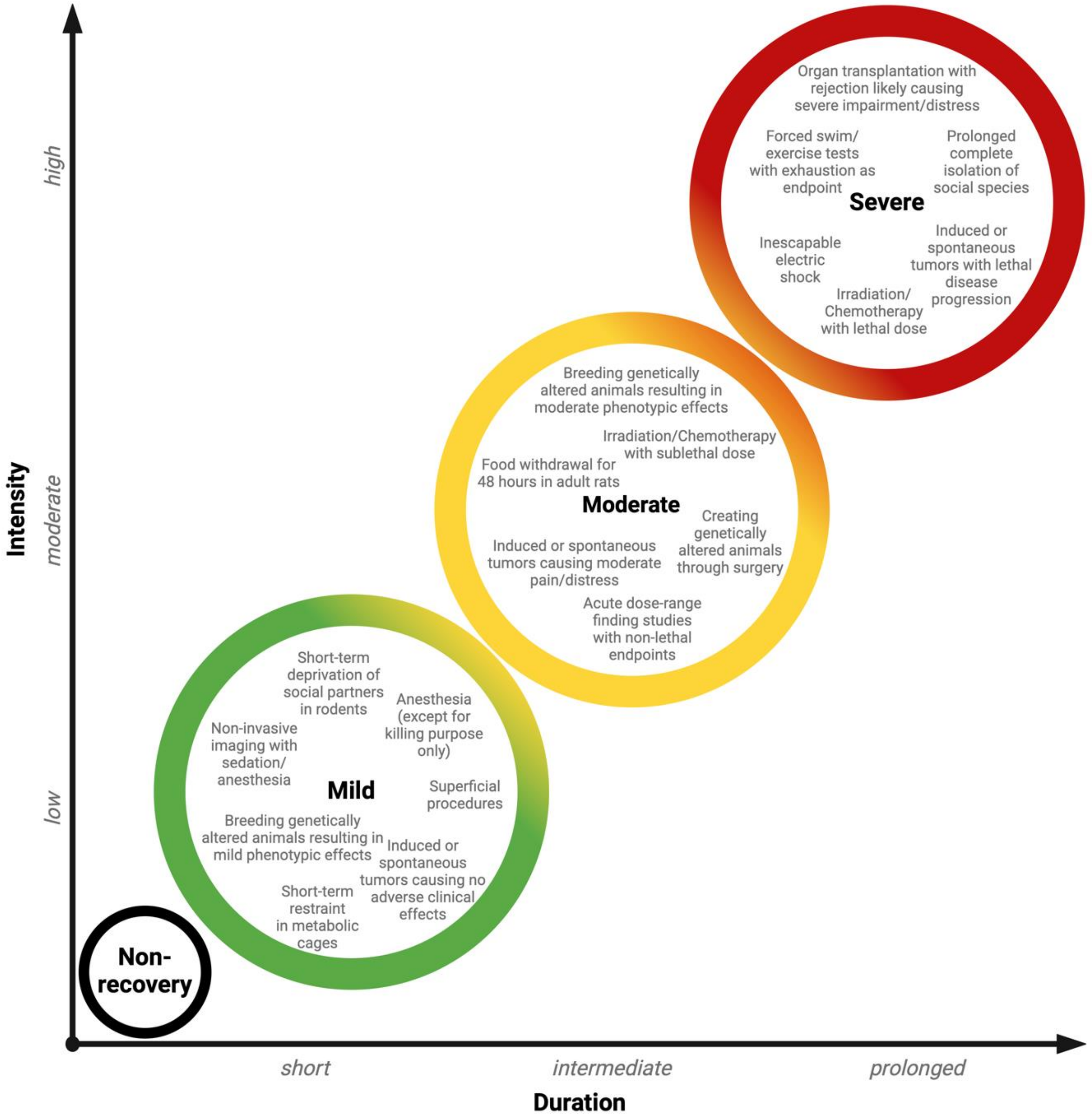

Project applicant

Instructions: Identify all harms linked to the procedures, assign each procedure with a corresponding number in the escalation model, place a bubble with a number within the escalation model, and list them accordingly.

List of all procedures for study group X:

1 ______________________________

2 ______________________________

3 ______________________________

4 ______________________________

etc. ______________________________

Please classify the severity for this study group by placing an "X" in the corresponding box below:

☐ Non-recovery ☐ Mild ☐ Moderate ☐ Severe

Committee member

Are the relevant harms adequately classified in the escalation model?

❑ Yes
❑ No

Has the severity of the study group been classified adequately?

❑ Yes
❑ No

B: Harm domain – Selecting the Severity Category of the Project (B.2)

Please select the severity category of the project based on the individual animal that is likely to experience the most severe effects (severity classification).

Note: The provided answer will feed into the decision matrix in the end.

Project applicant		Committee member	
Non-recovery	☐	Non-recovery	☐
Mild	☐	Mild	☐
Moderate	☐	Moderate	☐
Severe	☐	Severe	☐

B: Harm domain – Characterizing the Harm Domain (B.3)

Project applicant

When considering secondary modulating factors (see table below), is the harm domain on the upper or lower end of the severity category? Please indicate your answer with an "X" along the gradient for secondary modulating factors, after ticking the box for the severity category.

Please also indicate the animal number and animal percentage per severity category in the table below.

Secondary modulating factor	Description
**Animal's origin**	Animals originating from certain sources can indirectly affect animals through acclimation or habituation issues and/or pre-existing health conditions.
**Transportation**	Transport-induced health or stress (through transportation frequency and distance) indirectly affects animals before any procedure.
**Genetic modulations and their impact**	An indirect influence on an animal's physiology caused by phenotypic changes induced by genetic modifications.
**Species**	Selection of certain animal species can indirectly influence the harm domain, e.g., by using species that are known to have more or less sentience or cognitive abilities.
**Total number of animals**	An indirect factor that may influence the overall harm assessment.
**Duration in proportion to animal's lifespan**	The duration can indirectly influence the harm domain as its effect emerges rather cumulatively over the course of a project in relation to the animal's lifespan.

Severity category (SC)	Animal number per SC	Animal percentage per SC
**Non-recovery**		
**Mild**		
**Moderate**		
**Severe**		

Harm Domain Severity Category

☐ Non-recovery

☐ Mild

☐ Moderate

☐ Severe

Secondary Modulating Factors

Upper Limit

Lower Limit

Committee member

Have the modulating factors been adequately used to characterize the project harms within the selected severity classification?

❑ Yes
❑ No

C: Benefit domain – Describing the Anticipated Benefit (C.1)

	Project applicant	Committee member
	Please briefly state the project's anticipated benefit, by stating it below or referring to the relevant section of the project proposal:	Has the project applicant clearly stated the project's benefit?<br><br>❑ Yes<br>❑ No
	Please state who the beneficiaries are, and/or which areas will benefit, by stating it below or referring to the relevant section of the project proposal:	Has the project applicant clearly stated the project's beneficiaries?<br><br>❑ Yes<br>❑ No
Please assign the project's benefit to at least one of the following benefit streams.	❑ Social<br>❑ Socioeconomic<br>❑ Scientific<br>❑ Educational<br>❑ Safety/Efficacy testing	❑ Social<br>❑ Socioeconomic<br>❑ Scientific<br>❑ Educational<br>❑ Safety/Efficacy testing
What is the prospective timeline for the expected benefit to be achieved?	❑ Immediate benefit at the conclusion of the project.<br>❑ Delayed benefit at the conclusion of the project.	❑ Immediate benefit at the conclusion of the project.<br>❑ Delayed benefit at the conclusion of the project.

C: Benefit domain – Characterizing the Benefit Domain (C.2)

	Project applicant	Committee member
	To substantiate your selection, please refer to the relevant section in the project proposal:	
To what extent will the work's expected positive "real-world impact" affect humans, animals, and/or the environment?	❑ Negligible<br>❑ Low<br>❑ Significant<br>❑ Substantial	❑ Negligible<br>❑ Low<br>❑ Significant<br>❑ Substantial
To what extent will specific groups or areas benefit from this work?	❑ Negligible<br>❑ Low<br>❑ Significant<br>❑ Substantial	❑ Negligible<br>❑ Low<br>❑ Significant<br>❑ Substantial
What is the likelihood of achieving the benefit?	❑ Negligible<br>❑ Low<br>❑ Significant<br>❑ Substantial	❑ Negligible<br>❑ Low<br>❑ Significant<br>❑ Substantial
	**When considering the benefit domain as a secondary modulating factor for the outcome domain, to what extent does the benefit domain modulate the outcome domain? Please mark your answer with an "X" along the gradient for the benefit domain, after ticking the box for the outcome domain:**<br><br>Outcome domain: ☐ Low ☐ Intermediate ☐ High<br><br>**Benefit domain**: Negligible — Low — Significant — Substantial	Have the modulating factors been adequately used to characterize the benefit domain?<br><br>❑ Yes<br>❑ No

D: Justification domain – Summarizing the Results of the Harm-Benefit Analysis

Please copy the results from the previous harm and outcome domain evaluations into the decision matrix below by placing an "X" in the corresponding box, as shown on the first page. Cross-references to the relevant sections are made within the decision matrix. Please take into consideration the impact of secondary modulating factors on the harm and outcome domains.

Project applicant

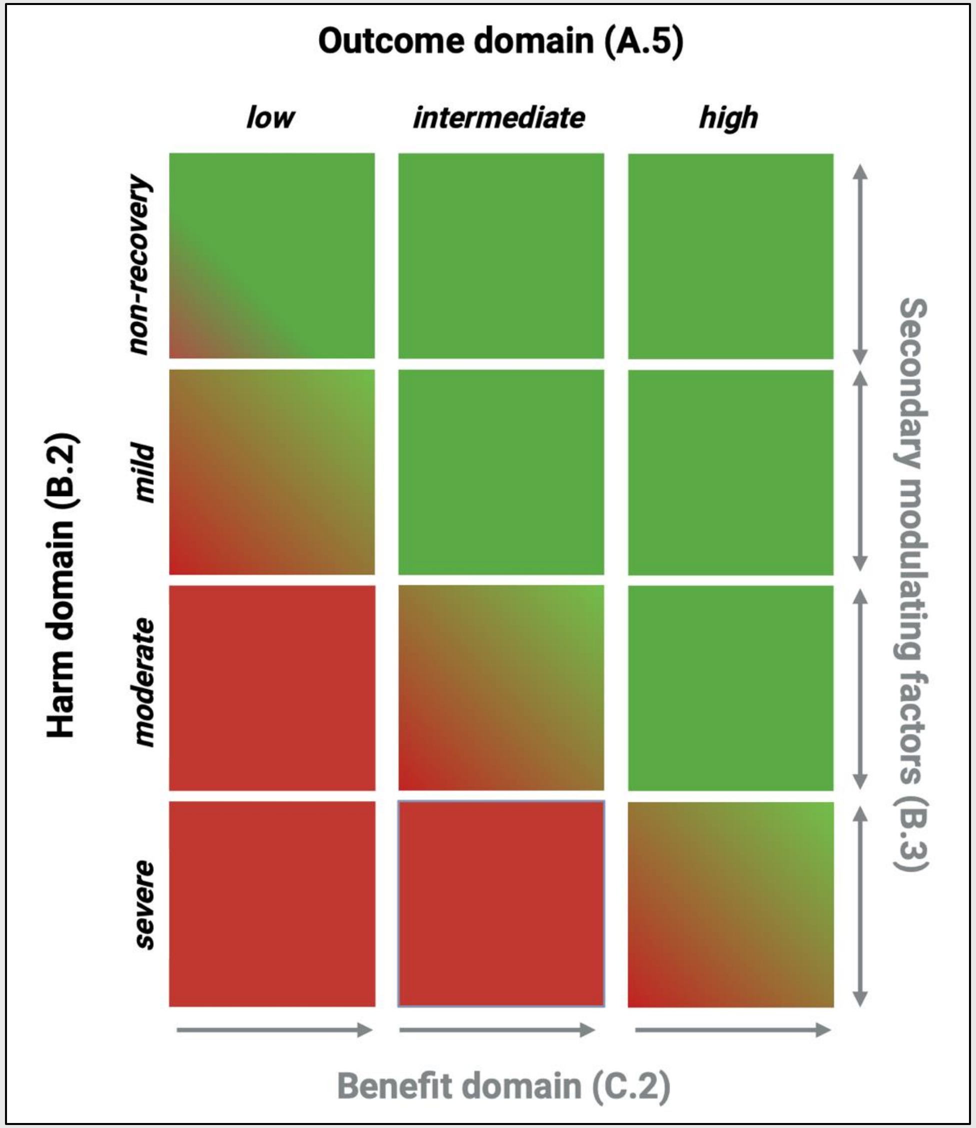

Committee member

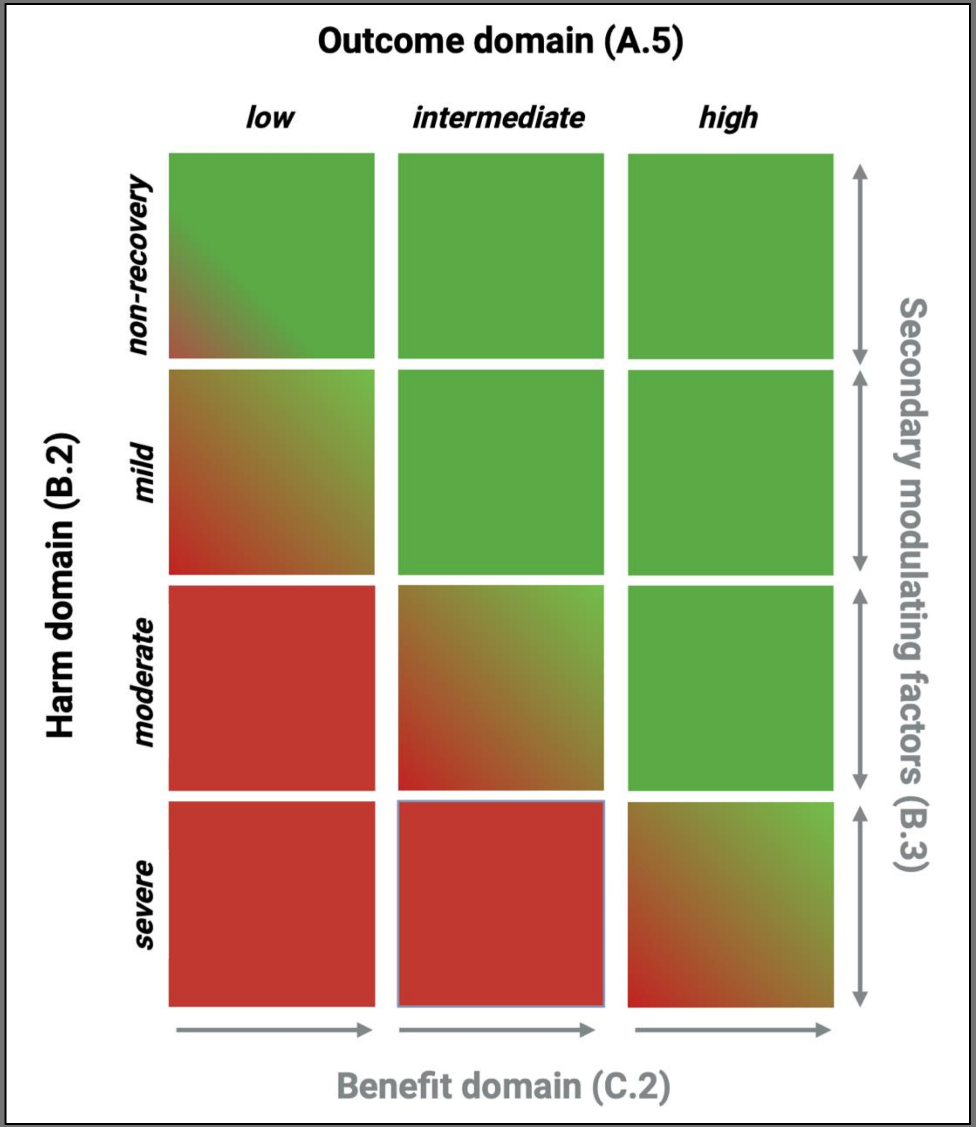

E: Documenting the Final Result

Project applicant

I attest that the result of the harm-benefit analysis, to the best of my reasoned discretionary judgment, is categorized as:

- ❑ Positive: The harm-benefit analysis speaks in favor of the project application.
- ❑ Negative: The harm-benefit analysis speaks against the project application.
- ❑ Pending: Further evidence is required to perform the harm-benefit analysis.

Date:

Name:

Signature:

Committee member

I attest that the result of the harm-benefit analysis, to the best of my reasoned discretionary judgment, is categorized as:

- ❑ Positive: The harm-benefit analysis speaks in favor of the project application.
- ❑ Negative: The harm-benefit analysis speaks against the project application.
- ❑ Pending: Further evidence is required to perform the harm-benefit analysis.

Notes:

Date:

Name:

Signature:
